# Streptococcus Infections of the Lungs in the British Army

**Published:** 1918-11

**Authors:** J. A. Wilson


VOL. II, NO. 4 PARIS NOVEMBER 1918

### WAR MEDICINE

Published by the American Red Cross Society in France
for the
Medical Officers of the American Expeditionary Forces

### RESEARCH SOCIETY REPORTS

### THE EIGHTH SESSION OF THE RESEARCH SOCIETY OF THE AMERICAN RED CROSS IN FRANCE

October 4 and 5, 1918, at the Hotel Continental, Paris.

*Major Walter B. Cannon, Chairman of the Research Society, presided at the first and second meetings of the Session, and Médecin-Major Rist at the third.*

*The subjects of the first meeting, Friday, October 4, at 2:00 P. M., were " Trench Foot " and " Tetanus ". Papers were presented by Médecin-Major J. Cottet, Service de Santé, Colonel F. H. S. Fuhr, C. M. G., D. S. O., B. A. in France, and Colonel Bailey K. Ashford, M. C., A. E. F. Major-General Sir David Bruce, C. B., F. F. R. S., presented the paper on Tetanus. Lieutenant-Colonel Joel Goldthwait, M. C., gave a demonstration of treatment of fractures of the humerus. (The papers read at this meeting will appear in the December number).*

*The subject of the second meeting of the Session, Saturday, October 5, at 9:30 A. M., was " The Restoration of the Sick and Wounded to the Line ". Papers were presented by Colonel Dalrymple, B. A., in France, Médecin-Major Laignel-Lavastine, Major Neff, M. C., and Major Alfred F. Cohn, M. C. (The papers read at this meeting will appear in the December number.)*

*The subject of the third meeting, Saturday, October 5, at 2:00 P. M., was " Streptococcus Infections of the Respiratory Tract ". Papers were presented by Captain J. A. Wilson, B. A. in France, Captain Robert L. Levy, M. C., Colonel Warfield Longcope, M. C., and Lt.-Colonel Joseph E. Capps, M. C.*

### STREPTOCOCCUS INFECTIONS OF THE RESPIRATORY TRACT

**Streptococcus Infections of the Lungs in the British Army.** Captain J. A. Wilson, B. A. F.

During the investigation of a severe epidemic of diarrheal diseases, a group of cases was encountered which presented relatively unusual features: firstly, in that the degree of anemia was quite out of proportion to the amount of blood present in the first few days in the feces, and secondly, in that the patient frequently developed the physical signs of pneumonia, a pneumonia which ran a prolonged course, was accompanied by a citron-colored sputum, and terminated by lysis.

1. *Clinical Features.* The individual cases gave a uniform history of sudden onset with pain over the abdomen, followed half an hour or so later by an urgent diarrhea, the feces containing a considerable admixture of blood, and in some cases, though not in all, of mucus. Tenesmus was a marked feature. The temperature rapidly rose to the vicinity of 102° or 103° F., and was associated with shivering, though no definite rigor was observed. The pulse was small and compressible, but it was not increased in rate in proportion to the rise in temperature. Most of the cases showed a herpes labialis.

The diarrhea persisted for from three to five days, when one of two things happened, either the patient made a complete and rapid recovery, or he developed the physical signs of a pneumonia — it is with the latter class we are concerned this afternoon.

The onset of the pneumonic state was sudden, and was ushered in by a rigor associated with a further rise in temperature of one or two degrees, but as in the diarrheal state the rise in pulse rate was not of a corresponding degree. The respirations became rapid and shallow, and the patient had the general appearance of being very ill. A further crop of herpes labialis was not an uncommon feature.

The patient complained of a feeling of discomfort, or of tightness over the whole chest, rather than of pain, but physical examination revealed nothing further than a certain harshness of the breath sounds with fine inspiratory crepitations.

Late on the second day there was evidence of a localization of the inflammatory process towards the base of one or other lung, pain being complained of in that region. By the third day there was definite dulness to percussion, and the breath sounds had become tubular. Fine crepitations were very abundant over this area.

At this stage of the disease the anemia became a pronounced feature of the case. The red corpuscles had dropped to about three and a half millions, while the hemoglobin stood at about 75 per cent. There was a slight leucopenia, affecting almost entirely the polymorphonuclear elements.

The sputum, at first scanty and tenacious, rapidly became blood-stained. By the third day it had become muco-purulent and relatively abundant, a citron-color being imparted to it by analysis of the contained blood.

The patient remained in such a state for a period of seven to nine days, when resolution of the lung lesions took place, the temperature falling by lysis. The sputum was very abundant and the return to the normal rapid and complete.

*Bacteriological Examination.* It was at first assumed that these cases were but anomalous forms of a paratyphoid or dysenteric infection, but the most thorough examination of the blood culturally and serologically, of the urine and feces culturally, could not adduce any evidence in support of this view. The examination of the feces microscopically and culturally brought out the fact that in all the cases there were very large numbers of streptococci; and that in those cases passing almost pure blood they were present in a great state of purity. It was considered, therefore, in the absence of other findings, that the streptococci were the cause of the diarrheal state, and it was determined to examine bacteriologically the sputum of the secondary pneumonia, in such cases, and also the blood, twenty-three cases being selected for the purpose.

In the examination of the sputum specimens were collected in sterile vessels at various stages of the disease. It was washed three times in normal saline, one part being used for the microscopic examination, a second for a cultural examination, and a third part for the inoculation of a mouse to exclude a pneumococcal infection.

The microscopical appearances were uniform. In the early stages the cellular elements were few, but by the third day of the pneumonic symptoms there was a large increase consisting mainly of polymorphonuclear and endothelial cells. The organisms were mainly of the streptococcal type; sometimes they were present in practically pure culture.

In the cultivation experiments, blood agar was used, and it gave very satisfactory results.

In no case did the inoculation of mice with an emulsion of sputum produce fatal issue.

For blood cultures 10° to 15° cc. of blood was withdrawn from a vein in the arm and distributed through three flasks, each containing about 50 cc. of citrated broth. The cultures were incubated for 48 hrs. at 37°C., the resultant growth, if any, being transferred to blood agar plates.

In twenty out of twenty-three cases examined, streptococci were obtained from the blood culture. It could, as a rule, be recovered up till the sixth day of the illness.

*The Characters of the Streptococci.* The organisms isolated from the sputum and blood appeared as minute translucent colonies at the end of 24 hours. There was a considerable variation in their hemolytic activity, but reference will be made to that feature later.

Some divergence was noted in the biochemical reactions. All strains produced acid glucose, lactose, saccharose, maltose, levulose, and salicin, while no change took place in dulcite, adonite, and sorbite. In mannite and inulin their behavior was erratic, some strains producing acid in one or other, the majority of the strains producing no change in either.

In litmus milk an acidity was produced which was followed in most cases by the formation of clot about the third day. Growth occurred on gelatin, but there was no liquefaction.

The hemolytic activities, as observed on the blood agar, were most variable, and no correlation could be determined between this property and the severity of the anemia or of the infection generally. It usually happened that a relatively non-hemolytic strain from the blood or the feces was associated with a very active strain in the sputum, yet from the immunological tests they appeared to be the same. A further point of interest was the fact that no relationship could be established between the hemolytic and the antigenetic powers.

The pathogenic properties of the strains were investigated in the mouse. Subcutaneous inoculation in the groin, as a rule, produced an abscess at the site of inoculation, but little evidence of constitutional change.

Intra-peritoneal inoculation, on the other hand, gave rise to an acute peritonitis, ending fatally on the second or third day, the organism being recovered from the pus, and in some cases, from the blood.

In such a widespread streptococcal infection — the organisms having been recovered from the feces, the lung, and in most cases the blood — it might be assumed that the blood reactions would be of a very definite character, but such was not found to be the case.

Agglutination reactions were carried out with heated and unheated broth cultures, the former at 56° C., the latter at 37°C., the macroscopic method being used in both instances. The heating of the cultures, which were 24 hr. growths, seemed to give more sharply defined results, and, judging from the microscopic examination of them, permitted of a more homogeneous emulsion being made. But, at the best, the results were unsatisfactory from the practical point of view.

| | | |
|---|---|---|
| One case gave an agglutination of | . . . . . . . | 1 : 250 |
| Six cases " " " | . . . . . . . | 1 : 125 |
| Four " " " " | . . . . . . . | 1 : 50 |
| Twelve " " no agglutination. | | |

The deviation of the complement tests carried out at the same period were not very satisfactory. Two types of antigen were used; the emulsions of the streptococci in normal saline in one case being heated at 56 C. for an hour; in the other they were used in their natural state. The standard of a positive result was the deviation of two minimum hemolytic doses of complement in addition to the amount deviated by the various components in the reaction. By this standard only four cases gave a positive result.

*Post Mortem Findings.* In this series of cases there were three which were fatal, death occurring on the second or third day after the onset of the pneumonic symptoms. The condition of the lungs at the post mortem examination was of special interest. They both showed a very acute congestion, the smaller bronchi being filled with a sanguineous, semipurulent exudation. Small areas of consolidation could be distinguished throughout both lungs, but at one or other base there was complete consolidation of the lower lobe. It was quite evident, however, that the lobar consolidation was really lobular, and was originally produced by the fusion of many broncho-pneumonic areas.

There was a mild degree of pleurisy over the lobe affected in this way.

*Conclusion*: Such were the findings in a series of rather interesting cases. From a consideration of them it would appear that streptococcal infections of the lungs are essentially broncho-pneumonic in their distribution, and that they are associated, as a rule, with septicemia which persists for two days or thereabouts after the onset of the pulmonary symptoms. Such infections do not appear to give rise to immune bodies as do many other infections, but it is quite probable that that is a question of technic.

There is just one further point which may be of interest and has to do with the possible origin of these cases. All the patients gave a history of having drunk shell-hole water, the diarrheal symptoms ensuing from one to five hours later. In an enquiry into the bacteriological flora of such waters it was found that in twenty-eight out of forty-two specimens examined streptococci were present in large numbers.

